# Emergence of carbapenem resistance in Gram-negative nosocomial bloodstream infections among critically ill children? A single-center experience

**DOI:** 10.1186/cc11775

**Published:** 2012-11-14

**Authors:** K Sasidaran, S Singhi, M Jaishree, A Bansal

**Affiliations:** 1 PGIMER, Chandigarh, India

## Background

Nosocomial bloodstream infections, especially by Gram-negative bacterial species, contribute to substantial morbidity and mortality in critically ill children. In this context, emerging carbapenem resistance further complicates patient management, as these isolates are often resistant to all β-lactam antibiotics and frequently co-resistant to most other antibiotics, leaving very few treatment options. We conducted this study to identify the microbiologic spectrum of Gram-negative nosocomial bloodstream infections (BSI) in a PICU of a tertiary care hospital and to determine the changing antibiotic sensitivity pattern.

## Methods

Data from prospectively maintained records of patients with more than 48 hours of PICU stay and a Gram-negative bloodstream isolate from January 2009 to December 2011 were analyzed. The study period was divided into Group 1 (January 2009 to December 2009), Group 2 (January 2010 to December 2010), and Group 3 (January 2011 to December 2011) to look for any change in antibiotic sensitivity pattern.

## Results

There were 102 episodes of Gram-negative BSI (Group 1: 51, Group 2: 33, Group 3: 18) in 1,601 patients, corresponding to 8.2 episodes per 1,000 patient-days. Among the Gram-negative isolates, commonest was *Acinetobacter *species (*n *= 44; 43%) followed by *Klebsiella pneumonia *(*n *= 16; 16%). All three groups were similar with respect to age, sex ratio, device utilization, grade of infection and outcome. When antibiograms were compared between Groups 1 and 3, there was a significant increase in carbapenem resistance (19% vs. 55%; *P *= 0.01) over time. Carbapenem-resistant isolates remained resistant to most of the other antibiotics (third-generation cephalosporins (98%), aminoglycosides (98%), β-lactam with β-lactamase inhibitor (94%), ciprofloxacin (90%)). However, sensitivity to colistin (100%) was retained in all carbapenem-resistant Gram-negative isolates. Although Gram-negatives were predominant organisms isolated in all three time periods, a significant increase in fungal isolates (3 vs. 12; *P *= 0.0003) was observed over the study period. All fungal isolates were candida species; nonalbicans candida species (13/19) were commoner than *Candida albicans*.

## Conclusion

Gram-negative bacteria are the commonest cause for nosocomial BSI in our PICU. An increasing trend of carbapenem (imipenam, meropenem) resistance was noted over the study period.

